# Analysis of some metallic elements and metalloids composition and relationships in parasol mushroom *Macrolepiota procera*

**DOI:** 10.1007/s11356-017-9136-9

**Published:** 2017-05-17

**Authors:** Jerzy Falandysz, Atindra Sapkota, Anna Dryżałowska, Małgorzata Mędyk, Xinbin Feng

**Affiliations:** 1 Laboratory of Environmental Chemistry & Ecotoxicology, Gdańsk University, 63 Wita Stwosza Str, 80-308 Gdańsk, Poland; 2 State Key Laboratory of Environmental Geochemistry, Institute of Geochemistry, Chinese Academy of Sciences, Guiyang, 550002 China

**Keywords:** Foraging, Fungi, Heavy metals, Trace elements, Mushrooms, Poland

## Abstract

The aim of the study was to characterise the multi-elemental composition and associations between a group of 32 elements and 16 rare earth elements collected by mycelium from growing substrates and accumulated in fruiting bodies of *Macrolepiota procera* from 16 sites from the lowland areas of Poland. The elements were quantified by inductively coupled plasma quadrupole mass spectrometry using validated method. The correlation matrix obtained from a possible 48 × 16 data matrix has been used to examine if any association exits between 48 elements in mushrooms foraged from 16 sampling localizations by multivariate approach using principal component (PC) analysis. The model could explain up to 93% variability by eight factors for which an eigenvalue value was ≥1. Absolute values of the correlation coefficient were above 0.72 (significance at *p* < 0.05) for 43 elements. From a point of view by consumer, the absolute content of Cd, Hg, Pb in caps of *M. procera* collected from background (unpolluted) areas could be considered elevated while sporadic/occasional ingestion of this mushroom is considered safe. The multivariate functional analysis revealed on associated accumulation of many elements in this mushroom. *M. procera* seem to possess some features of a bio-indicative species for anthropogenic Pb but also for some geogenic metals.

## Introduction

*Macrolepiota procera* (Scop.) Sing., commonly known as Field Parasol, Parasol Mushroom or Shaggy Parasol, is a saprobe. It is edible and widely collected in temperate regions and sub-tropical regions such as India, Thailand, China or Pakistan and across Europe (Kułdo et al. 2014; Melgar et al. 2016; Stefanović et al. 2016a; Širić et al. 2016; Xiaolan 2009). The pileus of *M. procera* are highly valued by locals. This is because of the taste and aroma of the cooked fresh individuals—sautéed, roasted, fried in butter or grilled, roasted with eggs or stuffed and broiled. According to some cooking recipes, the dried caps of *M. procera* could be resoaked in fresh water and both; the flesh and macerate (liquid) can be used for a dish. Frying of *M. procera* with butter or vegetable oil can to some degree result in leakage of elements out of a fleshy cap as was observed for fried *Cantharellus cibarius* and *Boletus edulis* and radiocaesium (^137^Cs) (Steinhauser and Steinhauser 2016). Nevertheless, caps of *M. procera* before frying are usually surrounded in flour, then in a drooping egg. Hence, any serious leakage of bio- or toxic elements out of a cap (or prepared dish) seems unlikely. Re-soaking of dried caps of *M. procera* in fresh water can have a more pronounced influence on possible leakage out of minerals but no figures are available. Blanching (parboiling) can decrease content of minerals in cooked mushrooms and also pickling, while a fate of a particular element can be different and highly dependent on its chemical form, localization within cells and type of chemical bonds made (Drewnowska et al. 2017a, 2017b; Falandysz and Drewnowska 2017).

*M. procera* prefers lighted and warm places. Especially in calcareous and sandy soils that are well-drained in forests, meadows and gardens (Rizal et al. 2015). In Asia, *Macrolepiota* species such as *M. procera*, *M. dolichaula* (Berk. & Broome) Pegler & R.W. Rayner, *M. gracilenta* (Krombh.) Wasser are consumed by locals (Woźniak 2009). In Europe, *M. procera* is mistaken with the deadly *Amanita phalloides* (Vaill. ex Fr.) Link., (Death Cap, or Destroying Angel) and *Chlorophyllum molybdites* (G. Mey.) Massee (False Parasol). Because of its popularity and versatility, it is also cultivated in kitchen gardens. This mushroom, like certain other macromycetes, when found in its natural habitats in background (unpolluted) areas, is efficient in accumulating toxic mercury (Hg), cadmium (Cd), lead (Pb), silver (Ag) and some micronutrients in fruiting bodies (Falandysz et al. 2001, 2003; García et al. 2009; Krasińska and Falandysz, 2016; Gąsecka et al. 2017; Melgar et al. 2009, 2016; Mędyk et al. 2017; Mleczek et al. 2013, 2016a, b, 2017; Saba et al. 2016a, b, c; Sarikurkcu et al. 2015). Due to its bioaccumulating property, many researchers are continuously investigating *Macrolepiota* species commonly collected by locals for their essential microminerals, macrominerals, metalloids and toxic metals contents in the fruiting bodies (Baptista et al. 2009; Falandysz et al. 2007a; Gucia et al. 2012a, b; Řanda et al. 2005).

This study attempts to investigate fruiting bodies of *M. procera* for its co-occurrence and associations between metallic elements and metalloids such as Ag, As, Ba, Be, Bi, Cd, Co, Cs, Cu, Ga, Ge, Hf, Hg, In, Li, Mo, Nb, Ni, Pb, Rb, Sb, Sn, Sr, Ta, Th, Ti, Tl, U, V, W, Zn, Zr and rare earth elements (Sc, Y, La, Ce, Pr, Nd, Sm, Eu, Gd, Tb, Dy, Ho, Er, Tm, Yb and Lu) accumulated in caps and stipes.

## Materials and methods

**Fig. 1:**
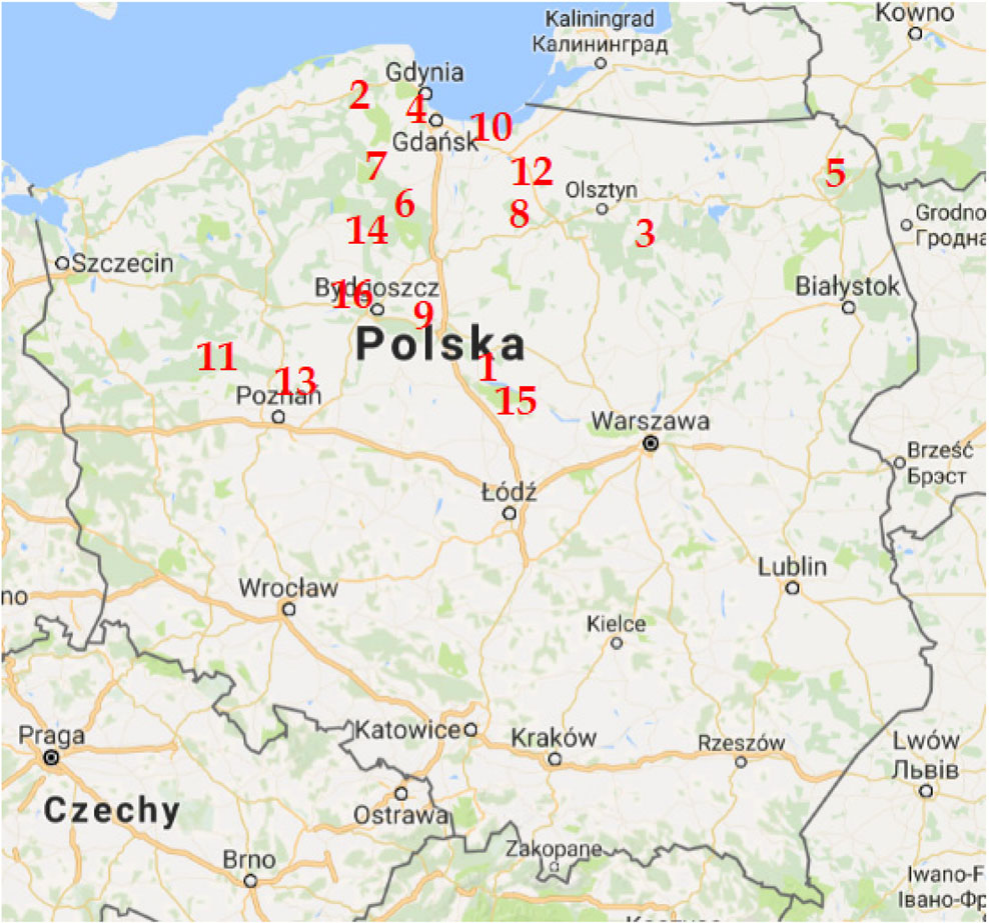
Sampling sites of *M. procera* (site 1: Włocławek—outskirts (forests), site 2: Pomerania, Lębork; site 3: Warmia land, Olsztyn/Szczytno; site 4: Trójmiejski Landscape Park—Gdańsk-Wrzeszcz; site 5: Augustów Primeval Forest; site 6: Tuchola Pinewoods, Łuby; site 7: Wdzydze Landscape Park; site 8: Warmia land, Sarnówek; site 9: Toruń—outskirts (forests); site 10: Vistula River Sand-bar, Stegna; site 11: Nadwarciańska Forest; site 12: Warmia land, Jeziorak lake—island of Gierszak; site 13: Zielonka near Poznań forests; site 14: Tuchola Pinewoods, Osie; site 15: Kukawy/Goreń region and site 16: Bydgoszcz forests; see also Table 1)

Fruiting bodies of *M. procera* were collected from 16 different sites from the lowland areas in northern and central regions of Poland: Włocławek - outskirts (forests) (52° 39′ 33″ N 19° 04′ 05″ E) [site 1; Fig. 1]; Pomerania, Lębork (54° 33′ N 17° 45′ E) [site 2]; Warmia land, Olsztyn/Szczytno (53° 47′ N 20° 30′ E/53° 33′ 46″ N 20° 59′ 7″ E) [3]; Trójmiejski Landscape Park—Gdańsk-Wrzeszcz (54° 22′ 10.1″ N 18° 35′ 47.0″ E) [site 4]; Augustów Primeval Forest (53*°* 87*′* 28*″* 0 N 22° 97′ 43″ 0 E) [site 5]; Tuchola Pinewoods, Łuby (53° 42′ 30″ N 18° 22′ 53″ E) [6]; Wdzydze Landscape Park (54° 00′ 47″ N 17° 54′ 04″ E) [7]; Warmia land, Sarnówek (53° 39′ 33.78″ N 19° 35′ 16.83″ E) [site 8]; Toruń—outskirts (forests) (53° 01′ 20″ N 18° 36′ 40″ E) [site 9]; Vistula River Sand-bar, Stegna (54° 19′ 35″ N 19° 6′ 44″ E) [10]; Nadwarciańska Forest (52° 12′ 00″ N 17° 54′ 00″ E) [site 11]; Warmia land, Jeziorak lake—island of Gierszak (53° 43′ 23.24″ N 19° 36′ 46.80″ E) [site 12]; Zielonka near Poznań forests (52° 33′ 13″ N 17° 06′ 49″ E) [site 13]; Tuchola Pinewoods, Osie (53° 35′ 57″ N 18° 20′ 41″ E) [site 14]; Kukawy/Goreń region (52° 33′ 52″ N 19° 11′ 42″ E/52° 31′ 50″ N 19° 17′ 22″ E) [site 15] and Bydgoszcz forests (53° 7′ N 18° 0′ E) [site 16] (Fig. 1, Table 1). The sites of *M. procera* collection can be considered as background (unpolluted) and without local or regional major emitters of heavy metals in forests of the lowland Poland. A major branch of metallurgy and ore mining industry is localized in the central (iron mill near Warszawa, Fig. 1) and southern regions of Poland (Brzezicha-Cirocka et al. 2016).

**Table 1:** Elements in fruiting bodies of *M. procera* (mg kg^−1^ dry biomass)

Place, year, number of specimens and morphological part^*^	Ag	As	Ba	Be	Bi	Cd	Co	Cs	Cu	Ga	Ge	Hf	Hg	In	Li	Mo
Augustów Primeval Forest, 2001 (*n* = 15; c) [5]^a^	1.9	0.93	5.4	0.019	0.0046	9.4	0.23	0.097	83	0.18	0.033	0.050	2.8	0.0011	0.23	0.45
Pomerania, Lębork, 2003 (*n* = 30; c) ltl [2]	1.4	0.69	1.7	0.0080	0.0021	2.4	0.31	0.034	130	0.12	0.022	0.022	2.5	0.0047	0.038	0.42
TLP, 2001 (*n* = 23; c) a [4]	0.86	0.47	2.5	0.0083	0.0058	0.75	0.084	0.039	57	0,13	0,025	0.020	1.9	0.0018	0.73	0.37
Vistula River Sand-bar, Stegna, 2003 (*n* = 10; c) [10]	0.98	0.43	10	0.021	0.0065	4.3	0.092	0.051	99	0.16	0.027	0.025	2.0	0.0023	0.48	0.49
WLP, 1994/2001 (*n* = 21; c) [7]	1.0	0.61	4.4	0.021	0.0064	1.8	0.18	0.041	96	0.18	0.040	0.049	1.1	0.0021	1.0	0.44
Warmia land, Gierszak, (*n* = 15; c) [12]	0.72	0.64	3.1	0.015	0.031	1.1	0.13	0.022	75	0.14	0.028	0.034	2.0	0.0029	0.094	0.39
Warmia land, Sarnówek, 2001 (*n* = 11; c) [8]	0.65	0.37	2.0	0.0056	0.0010	0.65	0.056	0.013	83	0.12	0.022	0.021	1.5	0.0017	0.49	0.35
Olsztyn/Szczytno, 2002 (*n* = 25; c) [3]	0.94	0.86	0.85	0.0086	0.0067	1.7	0.24	0.030	82	0.097	0.014	0.0045	1.9	0.0030	0.019	0.44
Tuchola Pinewoods, Łuby, 1995 (*n* = 15; c) [6]	2.5	1.3	3.3	0.013	0.0041	2.2	0.16	0.036	100	0.14	0.030	0.022	2.0	0.0027	2.7	0.42
Włocławek—outskirts (forests), 2004 (*n* = 15; c) [1]	4.1	1.0	3.7	0.021	0.0035	0.52	0.070	0.032	80	0.15	0.048	0.029	1.8	0.0052	0.074	0.72
Toruń—outskirts (forests), (*n* = 15; c) [9]	0.83	0.49	4.0	0.013	0.0019	1.3	0.062	0.045	81	0.15	0.036	0.018	2.8	0.0027	2.0	0.44
Nadwarciańska Forest, 1999 (*n* = 15; c) [11]	1.2	0.56	0.85	0.0088	0.0044	0.84	0.034	0.036	83	0.083	0.014	0.0032	2.2	0.0039	0.012	0.34
Zielonka near Poznań, 2001 (*n* = 15; c) [13]	7.9	5.4	2.5	0.023	0.0034	0.73	0.053	0.028	100	0.14	0.057	0.019	1.1	0.0035	0.094	1.4
*Mean*	1.9	1.1	3.9	0.014	0.0063	2.1	0.13	0.039	88	0.14	0.030	0.024	2.0	0.0029	0.62	0.61
*SD*	2.0	1.3	2.4	0.006	0.0073	2.4	0.09	0.020	17	0.03	0.012	0.014	0.5	0.0012	0.85	0.28
Tuchola Pinewoods, Osie, 2000 (*n* = 15; w) [14]	1.9	0.54	5.3	0.020	0.067	1.9	0.12	0.029	110	0.17	0.038	0.032	1.6	0.0036	0.90	0.38
Kukawy/Goreń region, 2001 (*n* = 15; w) [15]	1.1	0.66	2.2	0.0045	0.0043	1.1	0.13	0.041	110	0.11	0.024	0.015	1.4	0.0043	0.35	0.37
Bydgoszcz - outskrits, 2001 (*n* = 15; w) [16]	2.0	0.49	5.6	0.020	0.0047	1.0	0.18	0.037	93	0.19	0.043	0.030	1.3	0.0019	0.70	0.38
*Mean*	1.3	0.56	4.4	0.015	0.025	1.3	0.14	0.036	100	0.16	0.038	0.026	1.4	0.0033	0.65	0.38
*SD*	0.5	0.09	1.9	0.009	0.036	0.3	0.03	0.006	9	0.04	0.010	0.009	0.1	0,0012	0.28	0.01
Place, year, number of specimens and morphological part^*^	Nb	Ni	Pb	Rb	Sb	Sn	Sr	Ta	Th	Ti	Tl	U	V	W	Zn	Zr
Augustów Primeval Forest, 2001 (*n* = 15; c) [5]	0.058	0.42	6.1	50	0.057	0.054	1.4	0.018	0.040	32	0.063	0.014	1.0	0.012	63	2.1
Pomerania, Lębork, 2003 (*n* = 30; c) [2]	0.027	0.38	1.6	57	0.012	0.096	0.49	0.013	0.012	22	0.031	0.0057	1.3	0.015	65	1.2
TLP, 2001 (*n* = 23; c) [4]	0.047	0.21	2.2	58	0.011	0.25	0.73	0.015	0.022	27	0.024	0.0081	1.1	0.019	57	0.75
Vistula River Sand-bar, Stegna, 2003 (*n* = 10; c) [10]	0.058	0.39	4.6	38	0.020	0.18	1.4	0.016	0.035	39	0.021	0.016	1.1	0.017	69	1.2
WLP, 1994/2001 (*n* = 21; c) [7]	0.090	0.33	3.7	40	0.013	0.27	1.1	0.026	0.051	46	0.036	0.016	1.5	0.025	67	2.1
Warmia land, Gierszak, (*n* = 15; c) [12]	0.069	0.22	2.0	36	0.0095	0.28	0.87	0.010	0.017	33	0.023	0.010	1.3	0.019	54	1.4
Warmia land, Sarnówek, 2001 (*n* = 11; c) [8]	0.055	0.12	0.92	6.2	0.0068	0.14	0.70	0.015	0.023	27	0.0073	0.0071	1.1	0.017	60	0.93
Olsztyn/Szczytno, 2002 (*n* = 25; c) [3]	0.014	0.22	1.4	47	0.0075	0.19	0.26	0.012	0.0081	15	0.043	0.0027	0.84	0.018	54	0.17
Tuchola Pinewoods, Łuby, 1995 (*n* = 15; c) [6]	0.063	0.56	3.5	25	0.016	0.24	0.76	0.018	0.037	26	0.026	0.013	1.1	0.024	56	0.89
Włocławek - outskirts, 2004 (*n* = 15; c) [1]	0.068	0.26	2.8	20	0.026	0.13	1.3	0.017	0.023	34	0.013	0.010	1.6	0.040	92	1.3
Toruń - outskirts, (*n* = 15; c) [9]	0.061	0.18	3.3	34	0.012	0.17	1.1	0.021	0.025	29	0.045	0.010	1.2	0.025	58	0.83
Nadwarciańska Forest, 1999 (*n* = 15; c) [11]	0.009	0.53	2.0	15	0.0035	0.22	0.43	0.014	0.0043	13	0.0075	0.0019	1.1	0.0089	58	0.13
Zielonka near Poznań, 2001 (*n* = 15; c) [13]	0.042	0.26	2.8	10	0.021	0.30	0.94	0.0086	0.025	29	0.0066	0.010	3.3	0.038	150	0.91
*Mean*	0.051	0.31	2.8	33	0.017	0.19	0.88	0.016	0.029	29	0.027	0.0091	1.3	0.021	69	1.1
*SD*	0.023	0.01	1.4	17	0.014	0.07	0.37	0.005	0.013	9	0.017	0.0045	0.6	0.009	26	0.6
Tuchola Pinewoods, Osie, 2000 (*n* = 15; w) [14]	0.087	0.25	2.7	16	0.016	0.091	1.5	0.019	0.035	44	0.021	0.013	1.8	0.021	64	1.6
Kukawy/Goreń region, 2001 (*n* = 15; w) [15]	0.027	0.28	2.2	42	0.017	0.076	0.86	0.019	0.012	25	0.034	0.0058	1.6	0.016	64	0.70
Bydgoszcz - outskrits, 2001 (*n* = 15; w) [16]	0.085	0.35	2.0	19	0.019	0.21	1.9	0.017	0.052	33	0.030	0.019	1.1	0.025	51	1.3
*Mean*	0.066	0.29	2.3	26	0.017	0.13	1.4	0.018	0.033	34	0.028	0.013	1.5	0.021	60	1.2
*SD*	0.034	0.05	0.4	14	0.001	0.07	0.5	0.001	0.020	9	0.007	0.007	0.4	0.004	7	0.5

*c, *s*, *w* (caps, stipes, whole fruiting bodies, respectively); *TLP* Trójmiejski Landscape Park—Gdańsk-Wrzeszcz; *WLP* Wdzydze Landscape Park

^a^Localization of the sampling site (see Fig. 1)

Soils at the forested areas of the lowland Poland are podzolic soils which were formed by pine and mixed/pine forests and of mesophilic deciduous and coniferous forests in the zone of warm-temperate climate and are slightly acidic (Degórski 2004). Typical soils there are podzols, pseudopodzols and rusty soils poor in nutrients and developed from fluvioglacial sands with a texture of sands and somewhere in the outskirts of lakes and rivers with peats, peat-muck soils and vertisols. The tree covers are dominated by needle trees such as *Pinus sylvestris* L. and in lower proportion with *Picea abies* (L.) H. Karst., *Larix decidua* Mill., *Betula pendula* Roth, *Betula pubescens* Ehrh., *Alnus glutinosa* (L.) Gaertn., *Quercus robur* L., *Quercus petraea*, (Matt.) Liebl., *Fagus sylvatica* L. (Statistical Office 2014). Each composite sample of caps and whole fruiting bodies consisted of 10 to 30 individuals.

The fungal biomass dehydrated and grounded into a fine powder before analysis was dried at a temperature of 65 °C for 12 h and a subsample (about 200-mg samples made in duplicate) was mixed with 3 mL solution of ultrapure concentrated nitric acid (HNO_3_, 65%,) and 1 mL of ultrapure hydrofluoric acid (HF) in a polytetrafluoroethylene tubes (PTFE). Then, the tubes were screw tightened in stainless steel jackets and placed in an oven at 150 °C for 78 h. The solutions obtained were evaporated to dryness at 110 °C, to remove the excess of HF (Bi et al. 2007). Then, it was dissolved in 1 mL of HNO_3_ to make the final volume up to 50 mL, which was then transferred to a sample tube. As an internal standard, rhodium (Rh) (10–20 μg/L) was added to the samples prior to the Quadruple ICM-MS analysis (The Quadrupole-ICP-MS ELAN DRC-e; PerkinElmer, Waltham, MA, USA). In order to achieve good analytical quality control, quality assurance and blanks of certain certified reference materials were examined. Each element was measured three times and the values of relative standard deviation (RSD) were within 5% in the samples and the certified values for certified reference materials (CRM) (Liang and Grégoire 2000). The CRMs used were citrus leafs (GBW 10020) and soil (GBW 07405) produced by the Institute of Geophysical and Geochemical Exploration, China (Shi et al. 2011).

The computer software Statistica, version 10.0 (Statsoft Polska, Kraków, Poland), was used for statistical analysis of data and for graphical presentation of the results of two dimensional multiple scatter plot relationships between the variables.

## Results and discussion

### Toxic metallic elements and metalloids

Cadmium (Cd), mercury (Hg) and lead (Pb) are common constituents of *M. procera* and they occurred in caps at 2.1 ± 2.4 mg kg^−1^ db (arithmetic mean plus standard deviation) (Cd), 2.0 ± 0.5 mg kg^−1^ db (Hg) and 2.8 ± 1.4 mg kg^−1^ db (Pb) (Table 1). If assume that Cd, Hg and Pb remain in the flesh of caps, when they are sautéed, roasted, fried in butter, grilled or roasted with eggs, a single mushroom dish (100 to 300 g) certainly will provide an elevated quantity of each heavy metal (0.021–0.063 mg of Cd per capita, 0.02–0.06 mg Hg per capita and 0.028–0.084 mg Pb per capita). Hence, frequent eating of caps of *M. procera* could be not recommended. Nevertheless, unknown is the bioaccessibility of Cd, Pb and Hg contained in caps of *M. procera* for humans.

Contamination with toxic Cd and Pb of edible mushrooms is regulated in the European Union but not in the case of Hg, As or any other inorganic contaminant. The maximum limit of Cd established is 0.2 mg kg^−1^ fresh product (2.0 mg kg^−1^ in dried product—assuming moisture content is at 90%) in farmed *Agaricus bisporus* (J.E.Lange) Imbach, *Pleurotus ostreatus* (Jacq.) P. Kumm. and *Lentinula edodes* (Berk.) Pegler. This limit for Cd is 1.0 mg kg^−1^ fresh product (10 mg kg^−1^ in dried product) for other fungi (EC, 2006, 2008). In the case of Pb and cultivated mushrooms mentioned, the maximum allowed limit is 0.3 mg kg^−1^ fresh product (3.0 mg kg dried product) (EC, 2006, 2008). *M. procera* in this study showed at the average on little contamination with Cd, i.e., in caps, concentration levels were well below 10 mg kg^−1^ dried product (Table 1). An exception were individuals collected from the Augustowska Primeval Forest site which contained Cd in caps at 9.4 mg kg^−1^ dry biomass (Table 1). The Augustowska Primeval Forest region is considered as pristine (green lungs) and localized faraway of major emitters of heavy metals. A possible explanation for elevated concentration level of Cd in mushrooms can be because of a specific geochemistry of a soil parent material there, but this was not studied.

*M. procera* from the five sites contained Pb in caps at concentration level in the range of 3.3–6.1 mg kg^−1^ dry product (Table 1), which exceeded a limit set for farmed mushrooms mentioned earlier. Maximum contamination with Pb was similar to Cd in mushrooms from the Augustowska Primaeval Forest.

Also, silver (Ag) occurred in caps of *M. procera* at content comparable to what was observed for Cd, Hg, Pb, i.e., at 1.9 ± 2.0 mg kg^−1^ db. An intake of Ag per capita could be similar as is for Cd, Hg and Pb. Silver, like Cd, Hg and other chalcophile elements, has affinity to sulphur. The elements Ag, Cd and Hg are well bio-concentrated by *M. procera* and several other mushrooms (Chudzyński et al. 2011; Falandysz et al. 1994; Stefanović et al. 2016a, 2016b). Arsenic (As) was in caps at 1.1 ± 1.3 mg kg^−1^ db (Table 1), which was at relatively low concentration level while compounds of As were not studied. The inorganic compounds of As are most toxic while much less or almost non-toxic are considered organic arsenic compounds—they can be found in various (species-specific) proportion in mushrooms but can be well accumulated by fungi from a soil polluted with As (Falandysz and Rizal 2016; Falandysz et al. 2017a). There is no other data available on As in *M. procera* from background areas of Poland.

Available data on antimony (Sb) and thallium (Tl) in *M. procera* are scarce (Falandysz et al. 2001). In this study, Sb was in caps of *M. procera* at 0.017 ± 0.014 mg kg^−1^ db and Tl at 0.027 ± 0.017 mg kg^−1^ db, which are negligible quantities if compared to other toxic chalcophile elements mentioned earlier. In a view of the human consumer, there is a deficit of information on a possible absorption rate of a particular metallic elements and metalloids contained in cooked caps of this mushroom, when ingested. For example, the bioavailability of Cd from the blanched or pickled mushroom *Cantharellus cibarius* is considered to be not greater than 20% (unpublished, JF).

Other elements determined can be considered largely as natural compounds absorbed from the geochemical background that occurred at typical but not elevated concentration levels in *M. procera*. For example, the chalcophile elements determined such as gallium (Ga), germanium (Ge), indium (In), tin (Sn) and bismuth (Bi) were at the small contents in caps. They contained them (in mg kg^−1^ db), respectively, at 0.14 ± 0.03 (Ga), 0.030 ± 0.012 (Ge), 0.0029 ± 0.0012 (In), 0.19 ± 0.07 (Sn) and 0.0063 ± 0.0076 (Bi). A chalcophile copper (Cu) and zinc (Zn) were both the major trace elements in caps, which contained Cu at 88 ± 17 mg kg^−1^ db and Zn at 69 ± 26 mg kg^−1^ db (Table 1). Copper and zinc tend to accumulate similarly in the hymenophore and the rest of the fruiting body of *M. procera* (Alonso et al. 2003). Regardless of the contents of toxic elements such as Cd, Pb and Hg, the caps of *M. procera* seem a good source of Cu and Zn.

The alkali metals such as lithium (Li), rubidium (Rb) and caesium (Cs) were in caps at 0.62 ± 0.85 mg kg^−1^ db (Li), 33 ± 17 mg kg^−1^ db (Rb) and 0.039 ± 0.020 mg kg^−1^ db (Cs). For lithium there was a wide span of values for the sites and range from 0.012 to 2.7 mg kg^−1^ db (Table 1). There is no other data published on the element Li in *M. procera* to confirm observation from this study. Both Rb and Cs (stable ^133^Cs) were at a small content in *M. procera*, while much richer in both elements are mycorrhizal mushrooms (Falandysz and Borovička 2013). A low status of stable ^133^Cs (and also Rb) in fruiting bodies of *M. procera,* when related to certain other mushrooms, seem to explain a low susceptibility of this mushroom for contamination with radioactive caesium (^134/137^Cs).

The alkali earth metals such as beryllium (Be), strontium (Sr) and barium (Ba) highly differed in their content in *M. procera*. The element Be occurred in caps at 0.014 ± 0.006 mg kg^−1^ db, the Sr was at 0.88 ± 0.37 mg kg^−1^ db and the Ba was at 3.9 ± 2.4 mg kg^−1^ db. Data on Ba in *M. procera* provided in this study (Table 1) showed on a greater content, when compared to results for *M. procera* obtained by argon plasma atomic emission spectroscopy (Ouzouni and Riganakos 2007).

Other elements for which are available a few sets of data on their occurrence and accumulation by fungi in fruiting bodies are cobalt (Co), nickel (Ni), thorium (Th), titanium (Ti), uranium (U) and vanadium (V) (Aloupi et al. 2011; Baumann et al. 2014; Borovicka et al. 2011; Falandysz et al. 2007b; Vetter and Siller 1997; Řanda et al. 2005). Among mushrooms that were studied so far, the Fly Agaric *Amanita muscaria* (L.) Lam. was identified as the specific accumulator of vanadium, while not one specifically efficiently accumulated Co, Ni, Th, Ti or U. The caps of *M. procera* contained Co at 0.13 ± 0.09 mg kg^−1^ db, Ni at 0.31 ± 0.01 mg kg^−1^ db, Th at 0.029 ± 0.013 mg kg^−1^ db, U at 0.0091 ± 0.0045 mg kg^−1^ db, Ti at 29 ± 9 mg kg^−1^ db, and V at 1.3 ± 0.6 mg kg^−1^ db.

The obtained results for elements such as hafnium (Hf), which occurred in caps at 0.024 ± 0.014 mg kg^−1^ db, tantalum (Ta) at 0.016 ± 0.005 mg kg^−1^ db and wolfram (W) at 0.021 ± 0.009 mg kg^−1^ db. They all agree with a single result obtained for a whole fruiting body of *M. procera* from the Czech Republic and obtained by neutron activation analysis (Řanda and Kučera 2004).

Absent in the available literature are data on occurrence in *M. procera* of the metallic elements such as molybdenum (Mo), niobium (Nb) and zirconium (Zr). Those elements occurred in caps at 0.61 ± 0.28 mg kg^−1^ db (Mo), 0.051 ± 0.023 mg kg^−1^ db (Nb) and 1.1 ± 0.6 mg kg^−1^ db (Zr) (Table 1).

### Multivariate analysis of data

A possible relationship between 48 metallic elements (including data on rare earth elements) (Falandysz et al. 2017b) and metalloids accumulated in caps and whole fruiting bodies by fungus *M. procera* collected at 16 spatially distributed places in the northern and central regions of Poland has been examined using the principal component (PC) analysis (Wyrzykowska et al. 2001). In this multivariate approach, the results from examination of possible 48 × 16 data matrix are summarised in Table 2 (results for 48 × 13 data matrix obtained separately for caps are not shown). This was possible to explain up to 93% variability in the 48 × 16 data matrix by eight factors as well as up to 96% variability in the 48 × 13 data matrix by eight factors for which an eigenvalue value was ≥1. Absolute values of the correlation coefficient were above 0.72 (significance at *p* < 0.05) for 43 elements in the 48 × 16 data matrix and above 0.70 for 42 elements in the 48 × 13 data matrix.

**Table 2:** Factor loadings (Varimax normalized)

Eigenvalues	24.25	7.26	4.06	2.31	2.14	2.10	1.40	1.05
Total variance (%)	50.52	15.13	8.46	4.82	4.47	4.37	2.91	2.18
Cumulative %	50.52	65.65	74.11	78.94	83.40	87.77	90.68	92.86
Variables	PC1	PC2	PC3	PC4	PC5	PC6	PC7	PC8
Li	0.24	−0.16	−0.08	0.23	0.28	*0.72*	−0.12	0.09
Be	*0.76*	0.52	0,08	0.12	0.11	−0.08	0.00	−0.07
Sc	0.58	−0.08	0.18	0.08	0.25	−0.65	−0.07	0.00
V	0.01	*0.93*	−0.07	−0.09	−0.06	−0.01	−0.05	−0.24
Co	0.04	−0.20	0.23	−0.15	0.19	0.03	*0.84*	−0.11
Ni	0.02	−0.05	0.32	−0.07	*0.79*	0.19	−0.04	0.02
Cu	0.08	0.15	−0.07	−0.58	0.52	0.15	0.24	−0.37
Zn	−0.01	*0.95*	0.00	−0.05	0.05	−0.16	−0.12	0.03
Ga	*0.94*	0.13	0.22	0.10	−0.04	0.06	0.12	−0.03
Ge	0.63	*0.74*	−0.01	0.01	−0.11	0.10	−0.03	0.13
As	−0.15	*0.95*	0.05	0.16	0.12	−0.03	−0.06	−0.07
Rb	−0.11	−0.36	0.33	0.05	0.07	−0.19	0.70	0.23
Sr	*0.92*	0.08	0.14	−0.11	−0.07	−0.03	−0.19	-0.02
Y	*0.97*	-0.05	0.17	−0.03	0.03	−0.10	−0.06	0.03
Zr	*0.80*	0.10	0.23	0.01	−0.10	0.02	0.37	−0.18
Nb	*0.92*	0.02	−0.18	0.17	−0.12	0.18	0.03	−0.10
Mo	−0.02	*0.96*	0.00	0.06	0.06	−0.15	−0.08	0.09
Ag	0.06	*0.97*	0.00	−0.06	0.10	−0.01	−0.12	0.05
Cd	0.29	−0.12	*0.87*	0.01	0.18	−0.15	0.19	−0.10
In	−0.30	0.35	−0.12	*−0.77*	0.07	−0.10	0.11	0.04
Sn	−0.05	0.35	−0.13	*0.86*	0.10	0.05	0.06	0.08
Sb	0.44	0.24	*0.81*	−0.13	−0.06	−0.06	0.11	0.07
Cs	0.26	−0.08	*0.93*	0.00	0.08	0.01	0.13	0.13
Ba	*0.80*	−0.13	0.20	0.02	0.31	−0.27	−0.19	0.04
La	*0.92*	−0.01	0.26	−0.04	0.00	0.21	0.08	−0.15
Ce	*0.92*	−0.03	0.19	−0.02	0.01	0.25	0.09	−0.19
Pr	*0.92*	−0.03	0.22	−0.03	0.01	0.23	0.04	−0.18
Nd	*0.94*	−0.04	0.15	0.03	0.06	0.22	0.01	−0.15
Sm	*0.97*	−0.10	0.10	0.05	0.02	0.16	0.03	−0.07
Eu	*0.93*	0.15	0.12	−0.07	−0.15	0.11	−0.01	−0.12
Gd	*0.95*	−0.12	0.19	0.04	0.09	0.15	0.04	−0.01
Tb	*0.96*	−0.07	0.19	0.03	0.06	−0.02	0.00	−0.02
Dy	*0.95*	−0.08	0.22	0.06	0.09	−0.03	−0.10	0.01
Ho	*0.97*	−0.03	0.14	−0.05	0.00	−0.10	−0.06	0.05
Er	*0.98*	0.03	0.08	−0.04	−0.01	−0.11	−0.05	0.09
Tm	*0.93*	0.25	0.08	−0.05	−0.03	−0.17	−0.01	0.11
Yb	*0.97*	0.06	0.07	−0.10	−0.07	−0.13	−0.04	0.08
Lu	*0.96*	0.11	−0.01	−0.05	−0.06	−0.09	0.01	0.20
Hf	*0.78*	0.06	0.27	0.12	−0.12	0.03	0.37	−0.13
Ta	0.50	−0.33	0.19	−0.11	−0.06	0.62	0.04	0.08
W	0.37	*0.77*	−0.12	−0.03	−0.24	0.19	0.05	0.32
Tl	0.15	−0.28	0.66	0.04	−0.12	0.20	0.52	0.07
Pb	0.51	0.12	*0.73*	0.10	0.27	0.02	−0.01	0.09
Bi	0.33	−0.07	−0.19	−0.12	−0.13	−0.12	−0.03	**−** *0.76*
Th	*0.87*	0.05	0.13	0.25	0.13	0.30	0.05	-0.01
U	*0.93*	0.07	0.08	0.21	0.20	0.07	0.00	0.00
Ti	*0.90*	0.09	−0.07	0.05	−0.07	−0.01	0.08	−0.21
Hg	−0.06	0.16	−0.02	0.06	*0.89*	−0.13	0.18	0.13

In italics are the significant loadings used for each principal component

**Fig. 2:**
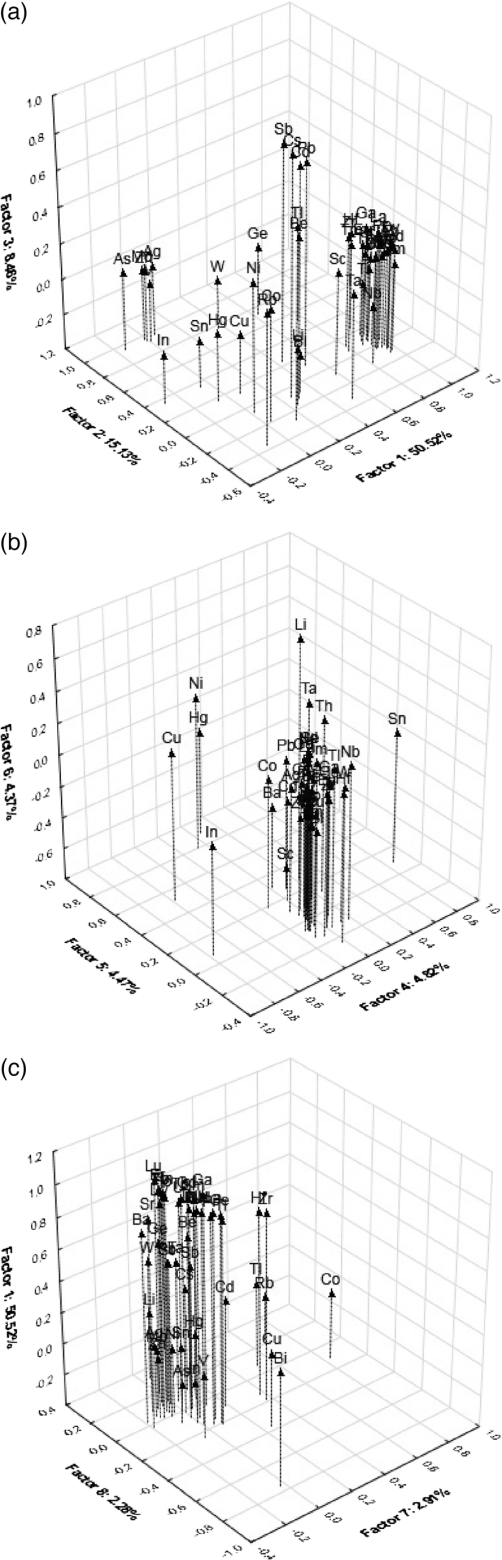
Principal component analysis of the trace metallic elements, metalloids and rare earth elements associations in *M. procera* mushroom (**a–c**) in the panorama of the Varimax normalized matrices

The PC1 was under influence by variables associated with positively correlated Ba, Be, Ce, Dy, Er, Eu, Ga, Gd, Hf, Ho, La, Lu, Nb, Nd, Pr, Sm, Sr, Tb, Th, Ti, Tm, U, Y, Yb and Zr, which are largely the lithophile elements that are characterised by similar chemical properties—alkaline earth metals (Be, Ba, Sr), which, together with Mg and Ca, have all a somewhat similar chemical and physical properties (Tabouret et al. 2010). The Be, Ba and Sr are more or less alike to Ca in the environment and biological systems and Sr can displace Ca. In the PC1 associations, positively correlated were also the rare earth elements (RREs) which are similar to Ca and all have similar chemical and physical properties and tend to exist together. The PC1 was also under the influence by variables associated with positively correlated some other elements (Y, Zr, Nb, U, Th, Ti) and also Ga. The PC2 was under the influence by positively correlated Ag, As, Ge, Mo, V, W and Zn, and PC 3 by variables with positively correlated Cd, Cs, Pb and Sb. The PC4 was influenced by variables associated with negatively correlated element indium (In) and positively correlated Sn, the PC5 was with positively correlated Ni and Hg, the PC6 was with Li, the PC7 was with Co and the PC8 with negatively correlated Bi (Table 2). The associations among the elements determined and places of mushroom collection in the factor space as a PCA are presented graphically in Figs. 1 and 2.

*M. procera* as a decomposer absorbs inorganic compounds from a digested decaying plant matter in soils and from the soil solution. Hence, a significant difference in content of the particular element in mushroom between the sampling localization could be largely associated with geochemistry of the soil parent material and content of a particular element and their availability or co-absorption, composition of decaying plant matter and anthropogenic pollution.

**Fig. 3:**
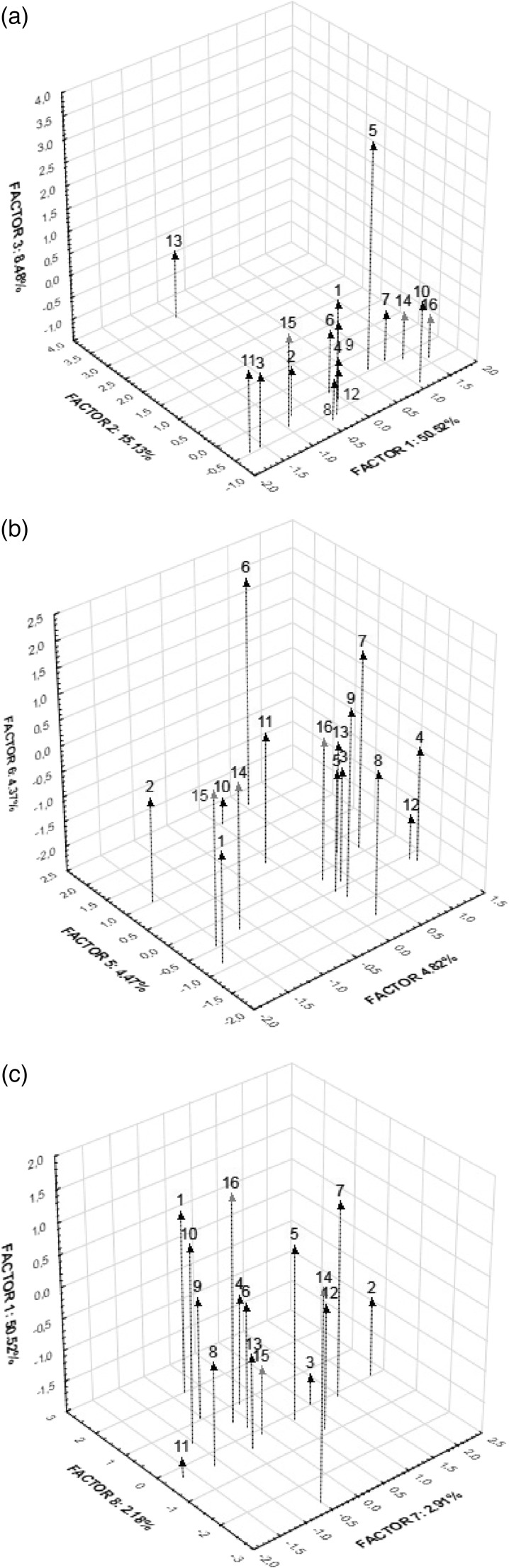
Principal component analysis of the trace metallic elements, metalloids and rare earth elements associations in its sampling localizations (**a–c**) of *M. procera* in the panorama of the Varimax normalized matrices

The localization Trzebiesza near Poznań—no. 13 on a map (associated with PC2) was separated due to significantly elevated content of Ag, As, Mo, V and Zn in *M. procera* (Figs 1a and Fig. 2a). Contrary, the localization Sarnówek in a forested and agricultural region of the Warmia land—no. 8 on a map (associated with PC 3) was separated due to small content of Cd, Cs, Pb and Sb in mushrooms (Fig. 2a and Fig. 3a). The localization of the Augustów Primeval Forest—no. 5 (associated with PC3) was characterised by elevated content of Cd, Pb and Sb, which could be related to known a deep in the ground deposits of some metal ores there. This localization was also associated with PC4 by small content of Sn and in mushrooms.

For the localization near Łuby in the Tuchola Pinewoods (no. 6) was strong relationship between Hg and Ni (associated with PC 5). The localization of Kościerzyna (no. 7) because of Li (PC6); the localization Lębork (no. 2), because of Co (associated with PC7), and the localization Island Gierszak (no. 12) because of Bi (associated with PC8) (Figs 1 and 2, Table 2).

## Conclusion

*M. procera* foraged from the background areas could be characterised by elevated content of toxic Cd, Hg and Pb in edible caps of the fruiting bodies while less of As, which is a species-specific feature. Since caps of *M. procera* are cooked without blanching, which could, to some degree, reduce the content of As, Cd, Hg and Pb, a frequent eating of this mushroom may be not desired. Also, toxic Sb and Tl were in *M. procera* at small but probably typical concentrations. *M. procera* seem to possess some features of a bio-indicative species for anthropogenic Pb but also for some geogenic metallic elements. The bio-elements Cu and Zn but also several other elements were in *M. procera* in a narrow range of concentration levels that can be explained by a lack of major environmental problems with heavy metals in the regions examined.
